# Development of a UPLC-MS/MS method for the determination of orelabrutinib in rat plasma and its application in pharmacokinetics

**DOI:** 10.3389/fphar.2022.991281

**Published:** 2022-09-06

**Authors:** Ya-nan Liu, Yingying Hu, Jing Wang, Chaojie Chen, Jianping Cai, Ren-ai Xu, Zhongqiu Lu

**Affiliations:** ^1^ Department of Pharmacy, The First Affiliated Hospital of Wenzhou Medical University, Wenzhou, Zhejiang, China; ^2^ Institute of Molecular Toxicology and Pharmacology, School of Pharmaceutical Sciences, Wenzhou Medical University, Wenzhou, Zhejiang, China; ^3^ Emergency Department, The First Affiliated Hospital of Wenzhou Medical University, Wenzhou, Zhejiang, China

**Keywords:** pharmacokinetic, UPLC-MS/MS, orelabrutinib, protein precipitation, methodological verification, rat

## Abstract

The aim of the present study was to establish an ultra performance liquid chromatography tandem mass spectrometry (UPLC-MS/MS) method for the determination of orelabrutinib in rat plasma using futibatinib as internal standard (IS), and to apply it for a pharmacokinetic study in rats. Orelabrutinib was extracted from plasma by protein precipitation and quantitatively analyzed by UPLC-MS/MS. An Acquity UPLC BEH C18 column was used for rapid separation by gradient elution using 0.1% formic acid and acetonitrile as mobile phases. The validation results of bioanalytical methodology showed that the linearity of orelabrutinib in plasma samples was good within the concentration range of 1–2000 ng/ml. The lower limit of quantification (LLOQ) was 1 ng/ml. The precision of orelabrutinib ranged from 1.4% to 11.5%, with intra-day and inter-day accuracy ranging from −5.7% to 7.7% and −0.2% to 12.5%, respectively. The selectivity, stability, matrix effect and recovery of the method all met the requirements of quantitative analysis of biological samples. The method was simple, sensitive, accurate and specific, and had high recovery rate. It also could be successfully applied to the pharmacokinetic study of rats.

## Introduction

Bruton’s Tyrosine kinase (BTK), belonging to the Tec family of non-receptor tyrosine kinases, is a membrane-binding protein that exists in all hematopoietic cells except T cells and natural killer cells (Herman et al., 2017; Ahn and Brown, 2021). BTK is an important signal molecule in the receptor pathway of B cells. It is expressed in all developmental stages of B cells and participates in regulating the proliferation, differentiation and apoptosis of B cells (Uckun, 1998). It plays an important role in the survival and proliferation of malignant B cells, and is a hotspot of clinical research on the treatment of B-cell tumors and B-cell immune diseases (Tsukada et al., 1993).

Compared with traditional therapies, BTK inhibitors are expected to be more effective and less toxic in treating a variety of B-cell malignancies and autoimmune diseases (Wang et al., 2019). Although ibrutinib, the first-generation BTK inhibitor, has been pioneering in the treatment of B-cell lymphoma, its adverse reactions are also worthy of attention, such as atrial fibrillation bleeding, diarrhea, rash, atrial fibrillation, and neutropenia (Davids and Brown, 2014; Paydas, 2019). From the current data, the reason for the interruption of treatment is often the intolerance of the adverse reactions of ibrutinib, rather than disease itself. To solve these problems, the researchers developed a new generation of BTK inhibitors, with high selectivity and specificity, to reduce off-target effects and adverse reactions (Burger, 2014; Shirley, 2022). Therefore, the development of the second generation of BTK inhibitors should improve the selectivity, drug resistance and reduce drug toxicity.

**FIGURE 1:**
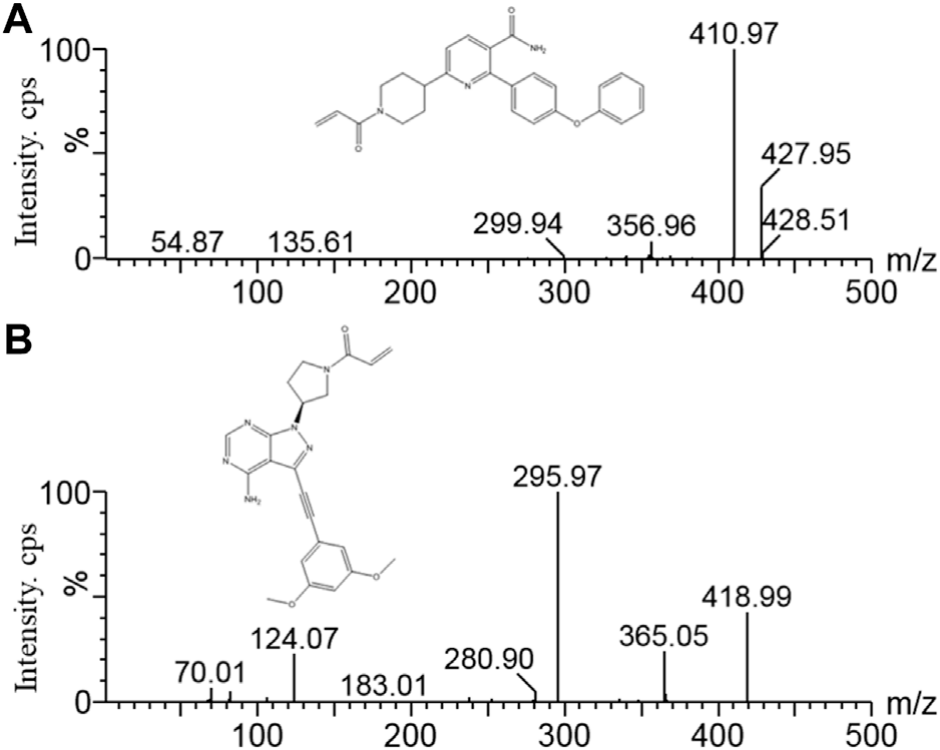
Mass spectrometry of orelabrutinib **(A)** and futibatinib (IS, **(B)**).

Orelabrutinib (Figure 1A) is a novel, powerful and irreversible second-generation BTK inhibitor independently developed in China, which was developed by InnoCare Pharma (Dhillon, 2021; Shirley, 2022). It is used to treat hematological malignancies such as chronic lymphocytic leukemia (CLL), small lymphocytic lymphoma (SLL) and mantle cell lymphoma (MCL), as well as autoimmune diseases such as systemic lupus erythematosus (SLE) and multiple sclerosis (MS) (Dhillon, 2021). On December 25, 2020, orelabrutinib was approved for the first time in China for the treatment of patients with MCL or CLL/SLL who have received at least one treatment in the past (China, 2021). Because of the importance of pharmacokinetic information for the optimization of clinical dosing regimens, it is necessary to monitor the concentration of orelabrutinib in plasma. At the same time, the ultra performance liquid chromatography tandem mass spectrometry (UPLC-MS/MS) bioanalytical method of orelabrutinib in plasma is unavailable.

In this experiment, we firstly established a UPLC-MS/MS method for the measurement of the concentration of orelabrutinib in plasma using futibatinib as internal standard (IS), which produced a comparable response and afforded high recovery. Through sample pretreatment and complete methodological validation, the method was demonstrated to be easy to operate, with high recovery, high sensitivity and specificity. The method was further successfully applied to the pharmacokinetic study of orally administered 10 mg/kg orelabrutinib in rats.

## Materials and methods

### Chemical materials and reagents

Orelabrutinib (purity >98%), futibatinib (purity >98%, used as internal standard, IS, Figure 1B), and formic acid (chromatographic grade), were provided by Beijing Sunflower Technology Development CO., LTD (Beijing, China). Acetonitrile (HPLC grade) and methanol (HPLC grade), were produced by Merck Company (Darmstadt, Germany). Ultrapure water was prepared by a Milli-Q water purification system produced by Milli-Q (Millipore, Bedford, United States).

### Preparation of standard curve and quality control (QC) samples

We precisely weighed an amount of orelabrutinib, and added diluent (methanol) into a measuring flask to dissolve it, and then obtained 1.00 mg/ml of orelabrutinib methanol stock solution after shaking. A series of standard working solutions (concentrations of 10, 50, 100, 500, 1000, 5000, 10,000 and 20,000 ng/ml) were obtained by gradient dilution with methanol. From the prepared standard working solutions, 10 μl was added to 90 μl of blank rat plasma to prepare calibration standards at concentrations of 1, 5, 10, 50, 100, 500, 1000 and 2000 ng/ml, and the standard curve was determined. The same method was used to obtain the lower limit of quantification (LLOQ) and three quality control (QC) samples (2, 200 and 1600 ng/ml). The appropriate amount of IS was also precisely weighed into the measuring bottle, and was shaked and dissolved to 1.00 mg/ml of the reserve solution with the solution of methanol. The reserve solution was continued to be diluted with methanol to obtain 500 ng/ml of the IS working solution. All ingredients and solutions were stored at −80°C for further use.

### Instrumentations and chromatographic conditions

Chromatographic analysis was performed using a Waters ultra performance liquid chromatography system consisted of a Waters Xevo TQ-S triple quadrupole tandem mass spectrometer (Milford, MA, United States) and a Waters ACQUITY UPLC I-Class system (Milford, MA, United States). An Acquity BEH C18 chromatographic column (2.1 mm × 50 mm, 1.7 μm) was used with the flow rate of 0.40 ml/min. In addition, other conditions were set as follows: injection volume of 1.0 μl, autosampler temperature of 10°C, column temperature of 40°C. The mobile phases were constituted of 0.1% formic acid in water (solution A) and acetonitrile (solution B) with a gradient elution as below: 0–0.5 min, 90% A; 0.5–1.0 min 90–10% A; 1.0–1.4 min, 10% A; and 1.4–1.5 min, 10–90% A. 90% A was then maintained at 1.5–3.0 min to reach equilibrium. The entire run time was 3.0 min.

A Waters Xevo TQ-S triple quadrupole tandem mass spectrometer was coupled with electrospray ionization (ESI) in positive ion mode for mass spectrometry. Measurements were conducted by multiple reaction monitoring (MRM), and the ion transitions for orelabrutinib and IS were *m/z* 427.95→410.97 and *m/z* 418.99→295.97, respectively. Data acquisition, data processing and instrument control were performed using Masslynx 4.1 software (Waters Corp, Milford, MA, United States).

### Pre-treatment of plasma samples

The proteins in the plasma were removed by protein precipitation, and the substance to be measured was extracted at the same time (Polson et al., 2003; Sun et al., 2021; Ye et al., 2021). 100 μl of plasma sample was accurately aspirated, and 10 μl of standard working solution of IS (500 ng/L) was added and vortexed for 1.0 min, then 300 μl of acetonitrile was added for precipitation. After vortexed for 1.0 min and centrifuged at 13,000 × *g* for 10 min at 4°C, the supernatant was finally obtained and aspirated into the autosampler bottle. Finally, only 1.0 μl of the sample was injected for analysis.

### Method validation

This test followed the FDA bioassay and the following parameters were examined: selectivity, calibration curve, LLOQ, accuracy and precision, matrix effect, recovery, and stability (U.S. Department of Health and Human Services) Food and Drug Administration, Bioanalytical Method Validation Guidance for Industry, 2018 (Accessed 19 June 2020) https://www.fda.gov/media/70858/download).

### Selectivity

The selectivity of the UPLC-MS/MS method was investigated by processing different batches of rat plasma samples. Blank plasma sample (no analyte, no IS), LLOQ plasma sample (with analyte and IS) and plasma sample at 1.0 h from a rat after administration were analyzed to assess the presence or absence of interference from plasma endogenous substances during the retention times of the analyte and IS.

### Calibration curve and LLOQ

Standard plasma was taken and processed according to the section of **Pre-treatment of plasma samples**. Using the concentration of orelabrutinib in plasma as the horizontal coordinate and the ratio of orelabrutinib to IS peak area as the vertical coordinate, a regression operation using weighted least squares was performed to obtain the calibration curve of orelabrutinib. LLOQ is the minimum concentration of the calibration curve, which should be accurate and precise within ± 20%.

### Precision and accuracy

To obtain inter-day and intra-day precision and accuracy, QC samples at three concentration levels (2, 200 and 1600 ng/L) were analyzed and each concentration was measured in five batches for three consecutive days. And individual calibrations were performed on each validation day. Relative standard deviation (RSD%) was used to express precision, and relative error (RE%) was used to express accuracy. A precision of 15% or less and an accuracy of ± 15% or less are considered acceptable.

### Matrix effects and recoveries

Orelabrutinib standard solution was added to the treated blank plasma and prepared at three concentrations (2, 200 and 1600 ng/L), and the peak areas obtained were compared with those obtained from pure standard solutions at three QC levels, which was the matrix effect (Matuszewski et al., 2003). The recovery was obtained by comparing the peak area before extraction of orelabrutinib with the peak area after extraction at the three QC levels (Xie et al., 2018).

### Stability

To investigate the stability of orelabrutinib in rat plasma, QC plasma samples of three concentrations were placed in different environments to examine the short-term, long-term, three freeze-thaw cycles and stability at 10°C. Short-term stability was determined by placing plasma samples at room temperature for at least 3 h. Long-term stability was determined by testing plasma samples after 21 days of storage at −80°C. The stability of the plasma samples was evaluated by measuring the stability of three freeze-thaw cycles from freezing (−80°C) to thawing (room temperature) three times. In addition, the stability was determined by storing the prepared plasma samples in an autosampler (10°C) for 4 h.

### Pharmacokinetic study in rats

Six male Sprague-Dawley (SD) Rats (weighing 250 ± 20 g) were obtained from the Laboratory Animal Center of The First Affiliated Hospital of Wenzhou Medical University (Zhejiang, China). They were housed in an environmentally controlled rearing chamber and given food and water that met the standard for 1 week. The animal experiment was approved by the Ethics Committee for the Protection and Use of Laboratory Animals of The First Affiliated Hospital of Wenzhou Medical University (Zhejiang, China).

The rats were fasted for 12 h before the start of the experiment and were given free access to water. Oral gavage was administered to rats at a dose of 10 mg/kg of orelabrutinib dissolved in 0.5% sodium carboxymethylcellulose (CMC-Na). Blood was collected at different collection points at 0.333, 0.667, 1, 1.5, 2, 3, 4, 6, 8, 12, 24 and 48 h after administration. After collected, blood in centrifuge tube was centrifuged for 10 min at 13,000 × *g* at room temperature, and 100 μl of plasma was separated and stored at −80°C prior to analysis. The concentrations of the analyte were determined using this validated UPLC-MS/MS method, while the non-compartmental model was analyzed using DAS 2.0 software (Drug and Statistical Software of the Shanghai Mathematical Pharmacology Professional Committee, China), and pharmacokinetic parameters were calculated.

## Results

### Selectivity

**FIGURE 2:**
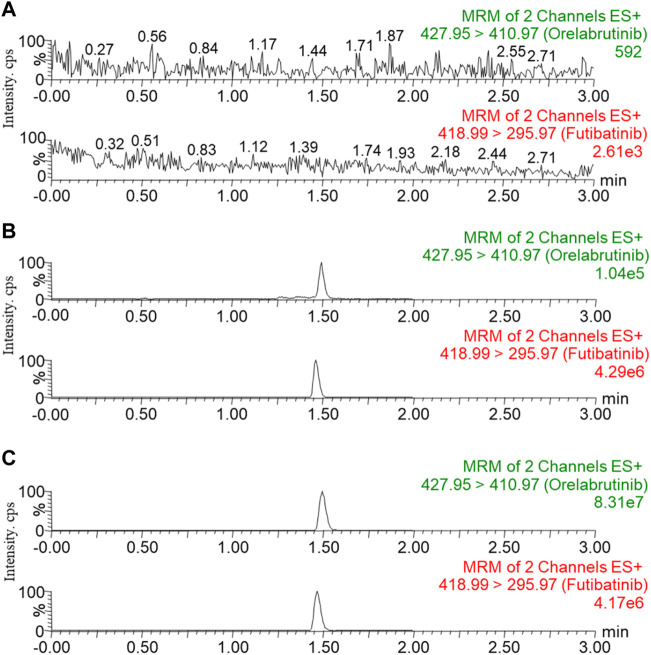
Representative MRM chromatograms of orelabrutinib and IS in rat plasma: blank rat plasma samples (**(A)**; no analyte, no IS); blank plasma samples spiked with LLOQ concentrations of analyte and IS **(B)**; plasma sample at 1.0 h collected from rats receiving a single dose of 10 mg/kg orelabrutinib **(C)**.

Six blank plasma samples from different rats were pretreated to detect the effect of matrix endogenous substances on the analyte and IS, and to test the selectivity of this method. Figure 2 showed the representative MRM chromatograms of blank rat plasma samples (A; no analyte, no IS), blank plasma samples with LLOQ concentration of analyte and IS added (B), and plasma sample at 1.0 h collected from a rat receiving a single dose of 10 mg/kg of orelabrutinib (C). The retention times for orelabrutinib and IS were 1.49 and 1.47 min, respectively. No interfering peaks were observed, indicating that the endogenous substances in this assay did not interfere with the analyte and IS.

### Calibration curve and LLOQ

The calibration curve had a good linearity in the concentration range of 1–2000 ng/ml. The linear regression equation for orelabrutinib was: Y= 0.0150887 × X - 0.0132206 (*r* ^2^ = 0.997). Its LLOQ was set at 1 ng/ml, and the actual concentrations calculated by the calibration curve relative to nominal values met the criteria of RSD% < 15% (Table 1, The RSD% at LLOQ <20%).

**TABLE 1:** Accuracy and precision of orelabrutinib in rat plasma (*n* = 5).

Analyte	Concentration (ng/ml)	Intra-day		Inter-day	
		Precision (RSD%)	Accuracy (RE%)	Precision (RSD%)	Accuracy (RE%)
Orelabrutinib	1	11.5	7.7	9.8	12.5
	2	4.4	−5.7	6.0	−0.2
	200	1.4	−3.3	4.8	2.0
	1600	1.5	−0.2	4.4	5.3

### Accuracy and precision

As shown in Table 1, the precision of orelabrutinib ranged from 1.4% to 11.5%, and the intra-day and inter-day accuracies ranged from −5.7% to 7.7% and −0.2% to 12.5%, respectively. Precision was met at less than 15%, and accuracy was within ± 15%, except LLOQ. The results were shown in Table 1 and were within the required range, indicating good precision and accuracy for the quantification of orelabrutinib in plasma.

### Matrix effects and recovery rates

Recovery and matrix effects were studied at three QC levels with plasma samples from different batch rats. As shown in Table 2, the recovery rates of orelabrutinib in rat plasma ranged from 95.5% to 100.2%. The matrix effect of orelabrutinib was calculated to be within the acceptable range (90.0–99.9%). Therefore, matrix effects had little effect on analyte ionization and did not affect the accuracy of the optimized UPLC-MS/MS method.

**TABLE 2:** Matrix effect and recovery rate of orelabrutinib in rat plasma (*n* = 6).

Analyte	Concentration (ng/ml)	Recovery rate (%)		Matrix effect (%)	
		Mean ± SD	RSD%	Mean ± SD	RSD%
Orelabrutinib	2	95.5 ± 2.7	2.9	90.0 ± 8.8	9.8
	200	100.2 ± 7.7	7.7	92.4 ± 6.5	7.1
	1600	97.8 ± 4.3	4.4	99.9 ± 8.5	8.5

### Stability

Stability experiments were performed to determine the stability of orelabrutinib in rat plasma under storage and analytical conditions. The stability of orelabrutinib in rat plasma was shown in Table 3. The results showed that orelabrutinib in plasma samples was stable after room temperature, 10°C, three complete freezing (−80°C)/thawing (RT) cycles, or storage at −80°C for 21 days.

**TABLE 3:** Stability of orelabrutinib in rat plasma under different conditions (*n* = 5).

Analyte	Concentration (ng/ml)	Room temperature			10°C			Three freeze-thaw			21 days		
		Mean ± SD	RSD (%)	RE (%)	Mean ± SD	RSD (%)	RE (%)	Mean ± SD	RSD (%)	RE (%)	Mean ± SD	RSD (%)	RE (%)
Orelabrutinib	2	2.1 ± 0.1	2.7	6.1	2.0 ± 0.1	3.4	−0.2	2.0 ± 0.1	5.8	1.0	2.1 ± 0.1	2.6	5.4
	200	199.3 ± 4.8	2.4	−0.4	208.0 ± 5.3	2.5	4.0	220.3 ± 8.5	3.9	10.2	212.6 ± 7.3	3.4	6.3
	1600	1603.9 ± 24.6	1.5	−0.2	1649.2 ± 25.3	1.5	3.1	1773.7 ± 57.6	3.2	10.9	1389.2 ± 147.3	10.6	−13.2

### Pharmacokinetic study in rats

**FIGURE 3:**
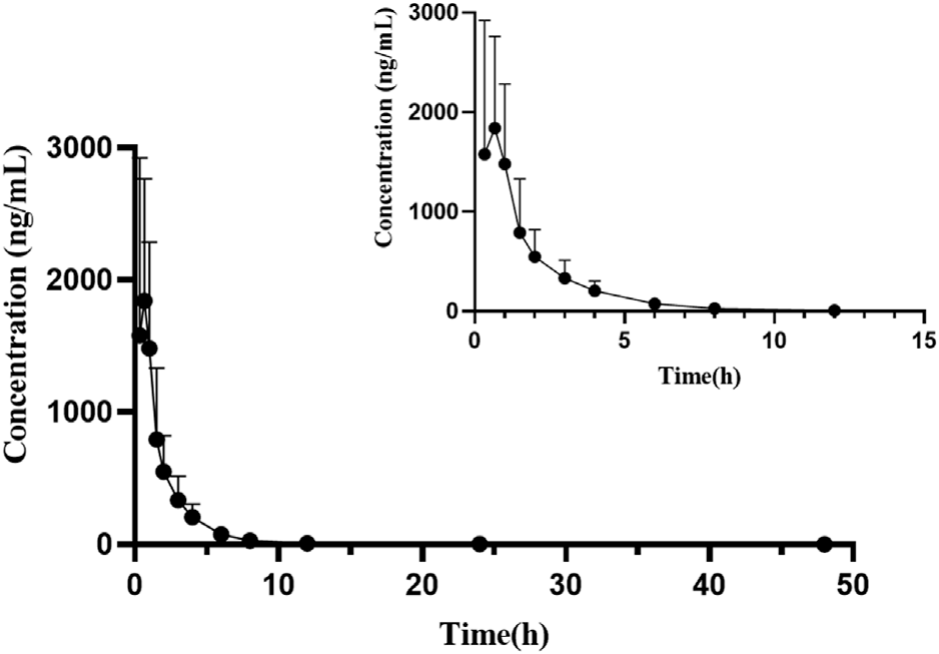
Mean plasma concentration-time curve of orelabrutinib after oral administration of 10 mg/kg orelabrutinib in rats (*n* = 6).

Pharmacokinetic studies were performed in rats using this newly developed UPLC-MS/MS technique, and the results of the changes in the concentration of orelabrutinib in rats after oral administration of orelabrutinib (10 mg/kg) were summaried. Figure 3 depicted the mean concentration of orelabrutinib in rat plasma (ng/ml) versus time (h), and Table 4 demonstrated the main pharmacokinetic parameters calculated in the non-compartmental model analysis.

**TABLE 4:** Main pharmacokinetic parameters of orelabrutinib in rats after a single oral dose of 10 mg/kg (*n* = 6, mean ± SD).

Parameters	Orelabrutinib
${AUC}_{0\to t}\left(ng/mL•h\right)$	3587.31 ± 1544.61
${AUC}_{0\to \infty }\left(ng/mL•h\right)$	3598.09 ± 1543.03
$t_{1/2}\left(h\right)$	8.25 ± 5.64
$T_{\mathrm{MAX}}\left(h\right)$	0.56 ± 0.17
${CL}_{z/F}\left(L/h\right)$	3.28 ± 1.44
$C_{\mathrm{MAX}}\left(ng/mL\right)$	1928.23 ± 1026.82

After oral administration of orelabrutinib to rats, it was rapidly absorbed and reached its maximum plasma concentration (T_max_) at 0.56 ± 0.17 h with a peak concentration (C_max_) of 1928.23 ± 1026.82 ng/ml and a half-life (t_1/2_) of 8.25 ± 5.64 h. In a phase 1 randomized dose-escalation study (NCT03189017; ICP-CL-001), the pharmacokinetic properties of orally administered orelabrutinib were evaluated in 64 healthy subjects who received orelabrutinib single (20, 50, 100, 200 and 400 mg) or multiple (100 or 200 mg once daily, 100 mg twice daily) doses for 14 days and showed that the AUC and C_max_ of orelabrutinib increased proportionally, indicating linear pharmacokinetics (Zhang et al., 2020). The C_max_ of orelabrutinib was only ∼300 ng/ml. Due to the individual differences among a small number of animals in this experiment (*n* = 6), the pharmacokinetic parameters of orelabrutinib need to be further studied to improve accuracy and confidence.

## Conclusion

In this experiment, a UPLC-MS/MS method was developed for the determination of a second generation BTK inhibitor orelabrutinib as the study target in plasma using futibatinib as IS. Through sample pretreatment and complete methodological validation, the method was demonstrated to be easy to operate, with high recovery, high sensitivity and specificity. The method was further successfully applied to the pharmacokinetic study of orally administered 10 mg/kg of orelabrutinib in rats. The results of this experiment are of great significance for the preclinical and clinical trials of orelabrutinib in future.

## Data Availability

The original contributions presented in the study are included in the article/Supplementary Material, further inquiries can be directed to the corresponding authors.
